# Litchi flower essential oil balanced lipid metabolism through the regulation of DAF-2/IIS, MDT-15/SBP-1, and MDT-15/NHR-49 pathway

**DOI:** 10.3389/fnut.2022.934518

**Published:** 2022-10-19

**Authors:** Yun Chen, Qiao Qin, Jingrui Luo, Yusi Dong, Chunxiu Lin, Houbin Chen, Yong Cao, Yunjiao Chen, Zuanxian Su

**Affiliations:** ^1^South China Agricultural University, Guangzhou, China; ^2^Guangdong Laboratory for Lingnan Modern Agriculture, Guangzhou, China

**Keywords:** litchi flower essential oil (LFEO), fat accumulation, lipid metabolism, energy metabolism, *Caenorhabditis elegans*

## Abstract

Many litchi flowers are discarded in China every year. The litchi flower is rich in volatile compounds and exhibits strong anti-obesity activity. Litchi flower essential oil (LFEO) was extracted by the continuous phase transformation device (CPTD) independently developed by our research group to recycle the precious material resources in litchi flowers. However, its fat-reducing effect and mechanism remain unclear. Employing *Caenorhabditis elegans* as a model, we found that LFEO significantly reduced fat storage and triglyceride (TG) content in normal, glucose-feeding, and high-fat conditions. LFEO significantly reduced body width in worms and significantly decreased both the size and number of lipid droplets in ZXW618. LFEO treatment did not affect energy intake but increased energy consumption by enhancing the average speed of worms. Further, LFEO might balance the fat metabolism in worms by regulating the DAF-2/IIS, *sbp-1/mdt-15*, and *nhr-49/mdt-15* pathways. Moreover, LFEO might inhibit the expression of the *acs-2* gene through *nhr-49* and reduce β-oxidation activity. Our study presents new insights into the role of LFEO in alleviating fat accumulation and provides references for the large-scale production of LFEO to promote the development of the litchi circular economy.

## Graphical Abstract

Litchi flower essential oil (LFEO) was extracted by the continuous phase transformation device (CPTD) independently developed by our research group to recycle the precious material resources in litchi flowers. Moreover, LFEO was recognized to reduce the fat accumulation and increase energy expenditure in Caenorhabditis elegans and its mediated fat-lowering mechanism was involved in DAF-2/IIS, SBP-1/MDT-15 and NHR-49/MDT-15 pathway. 
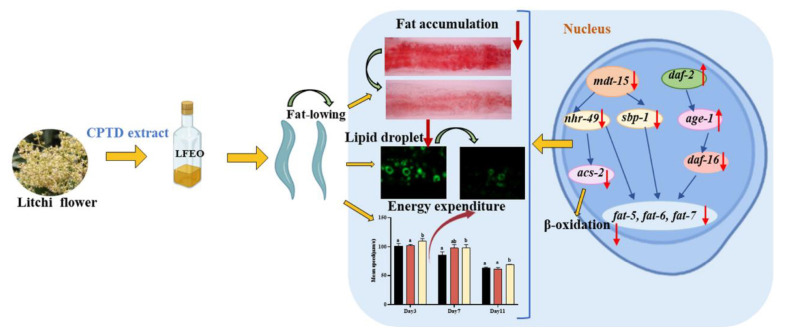

## Introduction

Obesity, with the high risk of diseases including cancer and diabetes, has become an epidemiological challenge worldwide. Nowadays, nearly one-third of the world’s population is overweight or obese (1). Furthermore, the World Health Organization predicts that nearly 60% of the worldwide population will be overweight or obese by 2030 (2). Obesity has various adverse effects on the body (3, 4) and poses a major public health threat (5). It increases the risk of a variety of diseases, such as diabetes, cardiovascular, cerebrovascular disease, and metabolic syndrome, which harm one’s quality of life and work productivity, and increase medical costs (3, 4). Five major anti-obesity drugs approved by the United States Food and Drug Administration, including orlistat, liraglutide, phentermine/topiramate, and naltrexone-bupropion, have been used in the treatment of obesity for many years (6, 7). However, these weight-loss drugs have potential long-term risks of side effects (6, 7). For example, the non-prescription drug orlistat was reported to have flatulence, steatorrhea, nephrotoxicity, hepatotoxicity, kidney stones, and pancreatitis as side effects (6, 7). Recently, some natural food ingredients, medicinal plants, and their bioactive components have gained attention from scientists because of their remarkable anti-obesity activity (8). Therefore, it is significant to explore natural products with fat-reducing biological activities.

Litchi (*Litchi chinensis Sonn*.), one of the tropical and subtropical fruit crops, has been cultivated for at least 2,000 years in China (9, 10). Since 2014, the annual output of litchi in China has exceeded 2 million tons, and the direct output value of planting litchi has reached more than 28 billion yuan (11). Litchi has the characteristics of a large panicle and abundant flowers, which consumes nutrition for flowering and finally results in fruit abscission (12). In order to increase the fruit yield of litchi, farmers need to prune large amounts of litchi inflorescence through mechanical flower thinning and chemical control technology to reduce nutritional consumption (12, 13). Therefore, large quantities of litchi flowers are discarded in the field every year, leading to environmental pollution. However, litchi flowers, containing abundant bioactive substances such as essential oil, have been proven to exhibit beneficial pharmacodynamic effects, such as regulating blood sugar and blood lipids, anti-tumor, and antioxidant (14–17). Researchers have confirmed that the extract of litchi flower exhibits strong anti-obesity properties (17–20). Therefore, discarding litchi flowers causes waste and environmental pollution, and it is vital to explore the valuable properties of litchi flower resources.

The continuous phase transformation device (CPTD) independently developed by our research group possess the advantages of a lower extraction temperature, lower pressure, and quicker time (21–23). So far, CPTD had been successfully applied to the extraction of finger citron essential oil and citrus peel essential oil and had a high extraction rate, zero solvent residue, simple subsequent treatment, and low production cost (21–23). More importantly, CPTD is well suited for large-scale industrial production (21–23). Consistent with the principles of “improving water quality through pollution reduction” and “by 2030, reduce waste generation through prevention, reduction, recovery and reuse” of the 2030 United Nations Sustainable Development Goals (SDGs) (24, 25), this study reduces waste and promotes a positive environmental impact via reusing, recycling, and recovering the valuable components in these litchi flowers with CPTD independently developed by our research group. Furthermore, in accord with the principles of SDG 8 (“decent work and economic growth”) and SDG 9 (“industry, innovation and infrastructure”) (24, 25), using CPTD for the efficient recycling litchi flower essential oil (LFEO) could promote the production of high value-added biological products, and increase jobs in farming, and indirectly promote agricultural productivity and sustainable food production system.

*Caenorhabditis elegans* is an important model organism in understanding fat metabolism and has the advantages of a simple structure, convenient observation, and clear fat synthesis and storage pathways (26). For fat synthesis in *C. elegans*, the acetyl coenzyme A, as a key substrate for fatty acid (FA) synthesis, is carboxylated by acetyl coA carboxylase to form malonyl coenzyme A which will be extended by fatty acid synthase to form various fatty acids which mainly contain palmitic acid (C16:0) (27). Some of the C16:0 can be integrated into triglycerides (TG) or phospholipids, and the other ones will be modified by fatty acid desaturase to form polyunsaturated fatty acids, which can be converted into TG stored in nematode lipid droplets. These lipid droplets can be stained and quantified with lipid-specific dyes such as Nile red, Oil Red O (ORO), or Sudan black since *C. elegans* is transparent (28). More importantly, the fat metabolism pathway is clear, and a large number of signaling pathways essential for regulating human lipid metabolism are conserved in *C. elegans* (29). For example, the nuclear hormone receptor (NHR) collaborates with MDT-15 to regulate the stearoyl-CoA-Δ9-desaturases (SCDs) FAT-5, FAT-6, and FAT-7 to adjust nematode lipid synthesis in both mammals and nematodes (27). Furthermore, nematode has a sterol response element binding protein (SREBP) homologous receptor protein SBP-1, which is not only an important regulator of fatty acid synthesis in this organism but also an important transcriptional regulator of fat in mammals (30). In all, *C. elegans* is an advantageous animal model to study FA modulation (28).

Overall, the study intends to employ *C. elegans* as the *in vivo* animal to determine the lipid-lowering effect of LFEO extracted by CPTD and elucidate the potential mechanism. Our research will present a new idea for recycling LFEO from litchi flowers in large-scale production and exploit the lipid-lowering properties of LFEO and promote the application of LFEO in natural fat-lowering products.

## Materials and methods

### Preparation of litchi flower essential oil and its determination of volatile components

The litchi flowers were first pruned and collected from the orchard (South China Agricultural University main campus teaching and research base, Guangzhou, China). They were then dried, crushed, and then sifted over 20 mesh. LFEO was extracted by the CPTD independently developed by our research group according to the previous protocols with some modifications (23). The extraction conditions were as follows: extraction temperature of 40°C, extraction pressure of 0.6 Mpa, extraction time was 90 min, analytical temperature of 50°C, and the solvent was n-butane. LFEO was obtained for further use.

The components of LFEO were analyzed by gas chromatography-mass spectrometry equipment (GC-MS) (7890B-7000D, Agilent Technologies, Palo Alto, CA, USA) with a non-polar HB-5 capillary column (30 m × 0.25 mm, 0.25 μm, Agilent Technologies, Palo Alto, CA, USA). LFEO was diluted 100 times with anhydrous ethanol before analysis with CG-MS. The oven temperature setting parameters were as follows: 50°C for 2 min, raised to 110°C at 10°C/min, gradually increased to 190°C at 4°C/ min, raised to 230°C at 5°/ min, and finally increased at 4°/ min to 280° and held for 5 min. The other GC-MS setup procedures were carried out as follows: the carrier gas was helium (99.999%) with a flow rate of 1.0 mL/min; injection volume of 0.5 μL; injection temperature of 250°; using electron bombardment of ion sources, electronic energy of 70 eV; ion source temperature of 280°; four pole temperature of 150°; solvent delay of 3 min; and quality scan range: 50–550 m/z.

### *Caenorhabditis elegans* strains

The wild-type *C. elegans* strain N2 (Bristol) and related mutants including XA7702 [*mdt-15 (tm2182)*], RB1716 [*nhr-49 (ok2 165)*], CB1370[*daf-2(e1370)*], TJ1052 [*age-1 (hx546) II*], GR1307 [*daf-16 (mgDf50) I*], and CE541 [*sbp-1 (ep79)*] were obtained from the *Caenorhabditis* Genetics Center. The mutants BX107 [*fat-5 (tm420) V*], BX106 [*fat-6(tm331) IV*], BX153 [*fat-7 (wa36) V*], BX160 [*fat-7 (wa36)/fat-5(tm420) V*], and BX110 [*fat-6 (tm331) IV/fat-5 (tm420) V*] and the *Escherichia coli* OP50 (*E. coli* OP50) were offered by Qinghua Zhou (Biomedical Translational Research Institute, Jinan University, Guangdong Province, China). In addition, ZXW618 [hkdIs618 (dhs-3p::dhs-3::gfp)] was kindly provided by Prof. Zhengxing Wu (College of Life Sciences and Technology, Huazhong University of Science and Technology, Hubei, China). Age-synchronized animals were obtained according to our previous method (31). Typically, eggs were incubated on NGM at a constant temperature of 20°, and then nematodes with L4 stage characteristics (abdominal dorsal surface with a crescent shape) were filtered for synchronization after 48 h of cultivation. The synchronized worms were moved to a new medium every other day during the propagation period. Treatments were carried out from the eggs, and worms were harvested for analysis after 60 h of administration unless expressly stated otherwise.

### Oil red O staining the liquid droplets

The ORO staining was performed following our previous approach (23). First, nematodes were collected into centrifuge tubes and washed twice with PBS. Next, nematodes were fixed in 4% paraformaldehyde after being anesthetized with 0.5% sodium azide in PBS. Following three freeze-thaw steps, 60% isopropanol was added to the samples for 20 min of dehydration. Finally, the freshly diluted ORO stain (4 mg/mL) was used to stain the nematodes for 12 h in the dark. A microscope (CX-41, Olympus Co., Tokyo, Japan) with a 40× objective lens was employed to record the stained worms. ImageJ software was utilized as a tool to measure and quantify the dye intensity. The experiments were repeated at least three times with more than 15 worms in each group.

### Determination of triglycerides in vivo

TG analysis was calculated according to our previous experiment (23). More than 2,000 worms were harvested and cleaned three times with PBS buffer. Following the manufacturer’s instructions, TG and bicinchoninic acid protein kits (Nanjing Jiancheng Bioengineering Institute, Nanjing, China) were utilized to analyze the worm’s extracts. TG content was standardized to calculate protein concentration. The extracts were examined at least three times.

### Quantitative analysis of lipid droplets in ZXW618

The analysis of label-free lipid droplet fluorescence in ZXW618 worms was conducted as previously described (32). Worms were narcotized and photographed with a confocal laser scanning microscope (LSM800, Carl Zeiss, Jena, Germany). Images were quantified by ImageJ software. There were three biological replicates with at least 15 worms per group.

### Measurement of body length, body width, and relative body surface

Worms were collected and subjected to body size determination on day 3 as described in the protocol in the previous experiment (23). The length of each worm from head to tail was defined as body length and the widest part of the body was described as body width. ImageJ software was used for body area analysis. Three independent biological replicates were performed in each experiment with 10 worms per group.

### Determining the movement speed of nematodes in WormLab

After exposure to LFEO for 3, 7, and 11 days, respectively, *C. elegans* was transferred to fresh plates and adapted for 1 min. Using WormLab VAS - enclosed video acquisition system (MBF Bioscience., Williston, VT, USA) the nematodes were recorded for 1 min. Further, the WormLab tracking system and software (MBF Bioscience, Williston, VT, USA) were employed to analyze the average speed of the worms using videos of the nematodes’ locomotion.

### Determination of pharyngeal pumping

The frequency of worm pharyngeal pumping was assessed by observing the motion of the terminal bulb of the pharynx over a 30 s period. At least 15 worms were tested in each group and three independent experiments were conducted.

### Reproductive capacity assay

After treatment, the synchronized L4 stage worms were transferred to a new plate and the number of offspring, including eggs and larvae, on the original plate was calculated every 24 h until production ceased. Hatchability was defined as the ratio of the number of offspring to the number of eggs laid. The experiment was repeated three times independently.

### Growing development assay

The number of nematodes in each developmental stage, including L2/L3, L4, and adults, was calculated under drug treatment 48 h after hatching. Each experiment was replicated three times independently and at least 100 individuals were recorded each time.

### *Escherichia coli* OP50 bacterial growth assay

The growth assay of *E. coli* OP50 bacteria was detected according to a previous protocol (23). *Escherichia coli* OP50 bacteria (OD600 = 0.4) were made into a liquid prepared with deionized water, ethanol [0.2% (v/v)], and LFEO solution (limiting density 30 μg/mL) and then cultured at 37°C with a shaker at 170 rpm. The OD 600 values were measured at hourly intervals over 12 h and the experiment was repeated three times.

### Gene expression analysis by quantitative reverse transcription-polymerase chain reaction

The quantitative reverse transcription-polymerase chain reaction (qRT-PCR) assay was conducted as described in published literature (32). Total RNAs were extracted with a TRNzol Total RNA Extraction kit (Tiangen Biotech Co., Ltd., Beijing, China) and complementary DNA was prepared by using the Tiangen Fast King RT Kit (With gDNase) (Tiangen Biotech Co., Ltd., Beijing, China). The qRT-PCR was performed using iTaq™ Universal SYBR^®^ Green Supermix (Bio-Rad Laboratories, Hercules, CA, USA) and LightCycler 480II fluorescence qPCR equipment (Roche Diagnostics, Basel, Switzerland). The relative expression of the genes was calculated via the 2^–ΔΔCt^ method with *act-1* as the reference gene. The data represented the average of the three independent tests and the primers are listed in Supplementary Table 1.

### Statistical analysis

The statistical analysis used was one-way analysis of variance (ANOVA) with Bonferroni tests as *post hoc* comparison through SPSS version 23.0 (IBM, Chicago, IL, USA). All graphs were drawn using GraphPad Prism 8 (GraphPad Software Inc., San Diego, CA, USA). All experiments were repeated three times, and consistent results were obtained from these independent experiments. Statistical data are expressed as means ± SD unless otherwise indicated. A *p*-value < 0.05 was considered statistically significant.

## Results and discussion

### The major composition of litchi flower essential oil

**FIGURE 1:**
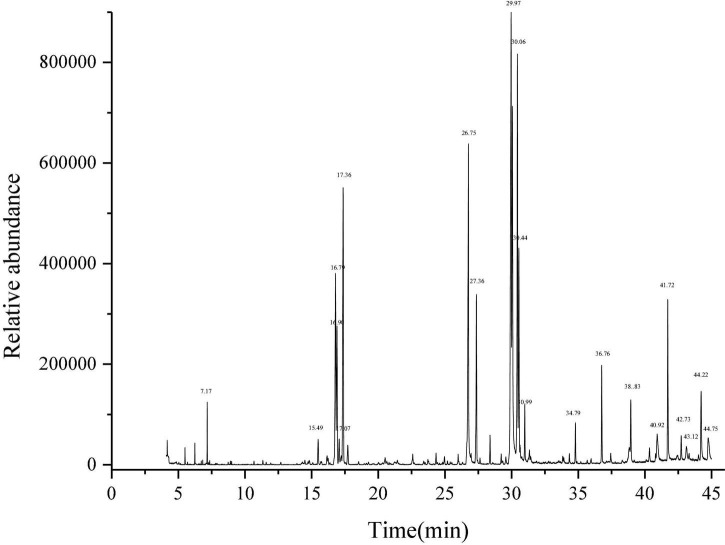
Determination of total ion chromatogram of essential oil of litchi flower by gas chromatography-mass spectrometry equipment (GC-MS) method.

Litchi flower essential oil was extracted by the CPTD independently developed by our research group (21–23). GC-MS method was used to identify the components of LFEO, and the relative content of each substance was determined by the peak area normalization method (33). As shown in Table 1 and Figure 1, 28 major compounds were identified, including alcohols, FA, esters, alkanes, and alkenes. Moreover, alcohol and FA compounds were the main components of LFEO, the contents of which were about 23.27 and 24.26%, respectively.

**TABLE 1:** Chemical compositions of volatile compounds of litchi flower essential oil (LFEO).

GC peak	Retention time	Volatile organic compound	Percent composition (%)
1	7.16	Tetraethyl silicate	0.695
2	8.90	Undecane	0.823
3	10.67	Dodecane	0.939
4	12.69	Tridecane	1.057
5	14.84	Tetradecane	0.957
6	16.79	α-Curcumene	9.656
7	16.90	Ylangene	0.838
8	17.06	Zingiberene	2.315
9	17.36	β-Bisabolene	7.304
10	17.71	β-Sesquiphellandrene	0.795
11	19.27	Hexadecane	0.933
12	21.42	Heptadecane	0.920
13	23.52	Octadecane	0.728
14	26.75	*n*-Hexadecanoic acid	6.874
15	27.36	Hexadecanoic acid, ethyl ester	1.129
16	29.94	Linoleic acid	9.753
17	30.03	α-Linolenic acid	7.629
18	30.43	Linolenic acid, ethyl ester	3.192
19	30.54	Ethyl alpha-linolenate	1.331
20	34.79	Phenol, 2,2′-methylenebis[6-(1,1-dimethylethyl)-4-methyl-	0.785
21	36.76	Bis(2-ethylhexyl) phthalate	3.353
22	38.86	Stigmasterol	4.876
23	38.94	Heptacosane	2.786
24	40.96	Gamma-Sitosterol	8.506
25	41.72	Nonacosane	3.590
26	43.13	Lupeol	1.951
27	44.22	Delta-tocotrienol	1.410
28	44.79	Hexacosyl heptafluorobutyrate	1.240

### Effect of litchi flower essential oil on triglycerides content in Caenorhabditis elegans

**FIGURE 2:**
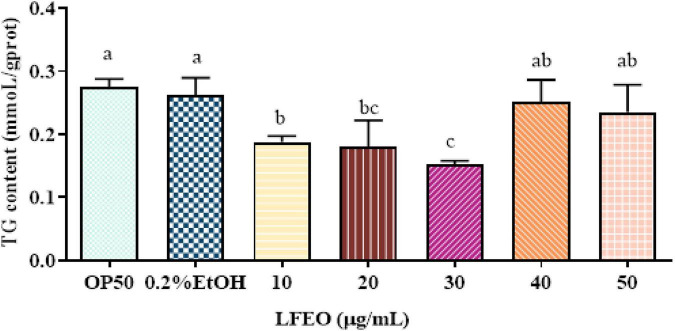
The effect of litchi flower essential oil (LFEO) on triglycerides (TG) content in different concentrations. Worms were fed with LFEO at different concentrations and cultured at 20°C for 60 h (*n* = three biologically independent samples; one-way analysis of variance (ANOVA) with Bonferroni tests as *post hoc*comparison; different letters indicate a significant difference, *p* < 0.05).

Obesity is mainly caused by the imbalance between the energy intake in food and the energy consumed (34). When energy intake exceeds energy expenditure, the excess energy will be stored in fat cells, which will enlarge the adipocytes and increase their quantity (35, 36). Moreover, it was reported that mammals store excess energy in adipocytes in the form of TG (37), and the main component of lipid droplets in *C. elegans* is TG (28, 30). Many studies show that plant essential oils are considered a class of potential anti-obesity substances (38, 39). Thus, to determine whether LFEO could reduce *C. elegans* fat aggregation, the effects of different concentrations of LFEO on TG content were examined. As shown in Figure 2 results reflected that the content of TG was significantly reduced at 10, 20, and 30 μg/mL LFEO treatment (*p* < 0.05), while no changes in the TG content were observed in LFEO treatment at 40 and 50 μg/mL (*p* > 0.05). In addition, there was a significant difference between the treatments of LFEO at 10 and 30 μg/mL (*p* < 0.05), but not between 20 and 30 μg/mL (*p* > 0.05, Figure 2). The results indicated that LFEO reduced TG accumulation in *C. elegans* and 30 μg/mL LFEO was the concentration used for further research.

### Litchi flower essential oil reduced the fat deposition in normal feeding, glucose feeding, and high-fat conditions

**FIGURE 3:**
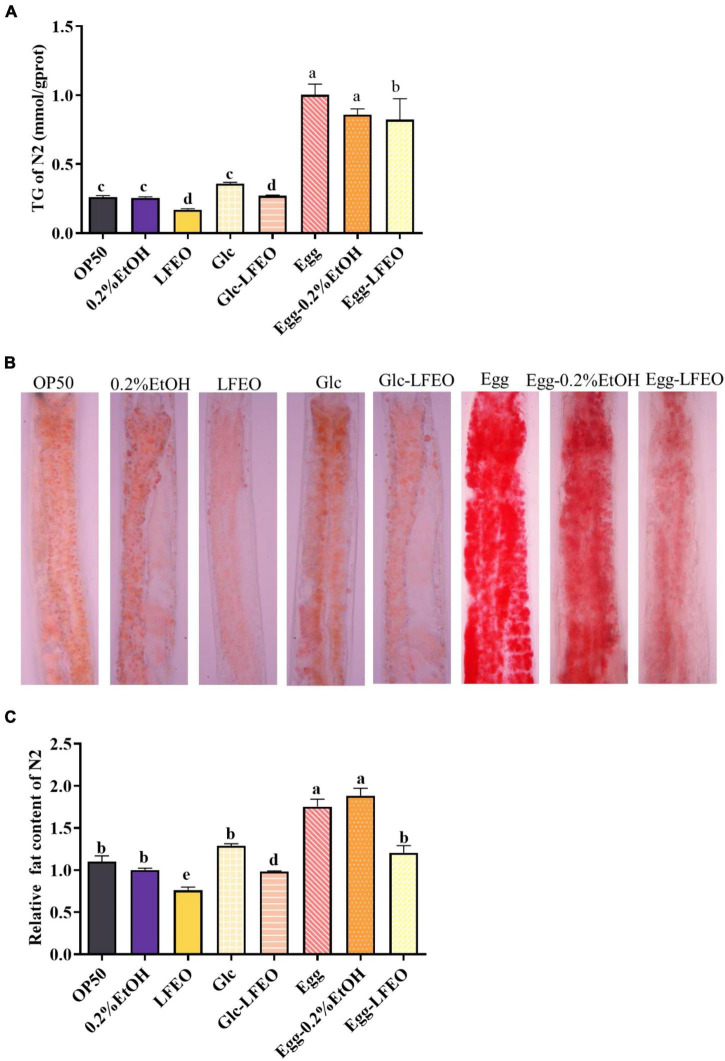
Effect of litchi flower essential oil (LFEO) on reducing fat accumulation in normal and glucose diet, and high-fat diet *C. elegans*. **(A)** Triglyceride contents of *C. elegans* incubated with LFEO in normal, glucose-feeding, and egg yolk feeding worms (n = three biologically independent samples; one-way ANOVA with Bonferroni tests as *post hoc*comparison; different letters indicate a significant difference, *p* < 0.05). **(B)** The representative pictures of nematodes stained with ORO. **(C)** Using image J software to quantify the pictures of ORO staining to calculate relative fat quantification of the worms (*n* ≥ 15 worms per group; one-way ANOVA with Bonferroni tests as *post hoc* comparison; different letters indicate a significant difference, *p* < 0.05).

Excessive lipids in adipocytes are associated with obesity and metabolic abnormalities, such as type 2 diabetes, cardiovascular disease, and ectopic lipid storage (1). Although *C. elegans* lacks the unique adipocytes of mammals, the fat of *C. elegans* is mainly stored in the intestinal tract and subcutaneous tissue in the form of small lipid droplets (28, 30). These lipids can be displayed by ORO staining and can be specifically attached to neutral fat molecules to color them an eye-catching red (28, 30). Thus, the ORO staining method was conducted to evaluate the influence of 30 μg/mL LFEO on the fat accumulation of the nematodes, which had a good correlation with TG content. Meanwhile, a 0.2% ethanol treatment of the nematodes was performed as a control group, and a group in which the nematodes were fed only *E. coli* OP50 was added to compare the accuracy of the experiment. Firstly, the normal feeding condition was considered, and the quantitative results of ORO staining showed that the lipid droplet content of nematodes was decreased by 23.80% after LFEO treatment (*p* < 0.05, Figure 3C). The content of TG significantly decreased by 35.03% (*p* < 0.05, Figure 3A). Secondly, to demonstrate the preventive role of LFEO in obesity with a high-carbohydrate diet, the experiment was carried out on glucose-fed worms and were administered both LFEO and glucose simultaneously for 60 h, starting from the eggs. As shown in Figure 3B, the ORO staining intensity of LFEO-treated worms was significantly lighter than those under a glucose-only feeding condition. Furthermore, the fat accumulation in LFEO-treated worms was significantly reduced by 23.84% compared to those under a glucose-only feeding condition in the quantification result of ORO staining (*p* < 0.05, Figure 3C). Furthermore, the TG content of LFEO-treated worms was also decreased by 24.80% in a glucose-feeding condition (*p* < 0.05, Figure 3A). Thirdly, to further investigate the effect of LFEO on fat accumulation in nematodes under a high-fat diet, the high-fat model was conducted using research from Wan et al. (40), feeding the nematodes with egg yolk. The results showed a significant increase in both TG content and ORO staining results in nematodes treated with egg yolk compared to those fed OP50, indicating that the egg yolk high-fat diet model was successfully established (*p* < 0.05, Figures 3A,C). Moreover, LFEO significantly reduced the lipid content of high-fat diet nematodes in both the TG test and ORO staining (*p* < 0.05, Figures 3A,C). All the above results showed that LFEO could significantly reduce the fat accumulation of nematodes in normal feeding, glucose feeding, and high-fat diet conditions.

### Litchi flower essential oil reduced DHS-3::gfp-marked lipid droplet size and amount in *Caenorhabditis elegans*

**FIGURE 4:**
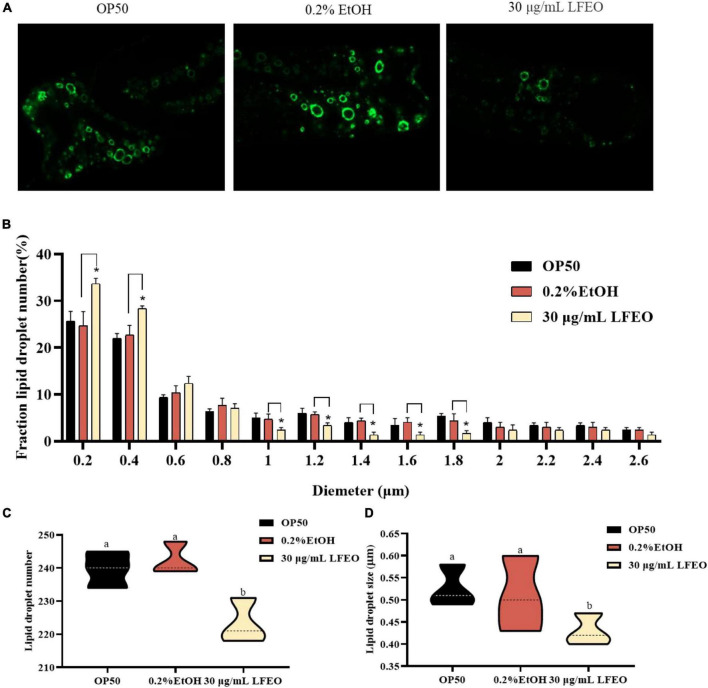
Effect of litchi flower essential oil (LFEO) on the number and diameter of lipid droplets in mutant ZXW618. **(A)** Representative fluorescence images of ZXW618 worms treated without or with 30 μg/mL LFEO. **(B)** Distribution of different size lipid droplets in ZXW618. **(C)** Average number of lipid droplets in ZXW618. **(D)** Average diameter size of lipid droplets in ZXW618. Statistical analysis of the results was done using a one-way ANOVA with Bonferroni tests as *post hoc*comparison; different letters indicate a significant difference, *p* < 0.05, with at least 15 worms per group.

Lipid droplets act as ubiquitous fat storage organelles that consist of a neutral lipid core, containing TG and sterol esters, surrounded by a phospholipid monolayer membrane with several decorating proteins (41). Short chain dehydrogenase (DHS) is almost entirely localized on the lipid droplet of *C. elegans*, which implies that DHS-3 could serve as a lipid droplet marker protein (42). In a DHS-3::gfp transgenic strain ZXW618, green fluorescent protein can visualize the lipid droplet marker protein DHS-3 (42). Therefore, to further investigate whether LFEO affected the regulation of lipid droplets, the mutant ZXW618 was used to observe the distributions of lipid droplets. The quantitative result displayed an obvious difference in the distribution of lipid droplets in ZXW618 worms between the LFEO treatment and control groups (Figure 4A). First, the section of small-size lipid droplets ranging from 0 to 0.4 μm was significantly increased, and the section of big-size lipid droplets ranging from 1 to 1.8 μm was significantly decreased in the LFEO group (*p* < 0.05, Figure 4B). Second, compared to the control group, the LFEO treatment markedly decreased the average number of lipid droplets in worms (*p* < 0.05, Figure 4C). Moreover, as shown in Figure 4D, the average size of lipid droplets of LFEO treatment was 0.43 μm, which was 15.7% less than that in the control treatment group (*p* < 0.05). This finding was in accordance with the research that litchi flower-water extract has the potential to decrease the cell sizes of adipose tissues and shows strong anti-fat activity (19, 20). Moreover, when energy is required, the neutral fat in the lipid droplets is decomposed into FA which can be used for energy production via β-oxidation (37). Therefore, we found that LFEO decreased the number and size of lipid droplets in *C. elegans* and might reduce fat accumulation via enhancing energy metabolism.

### Litchi flower essential oil influenced the energy metabolism of Caenorhabditis elegans

**FIGURE 5:**
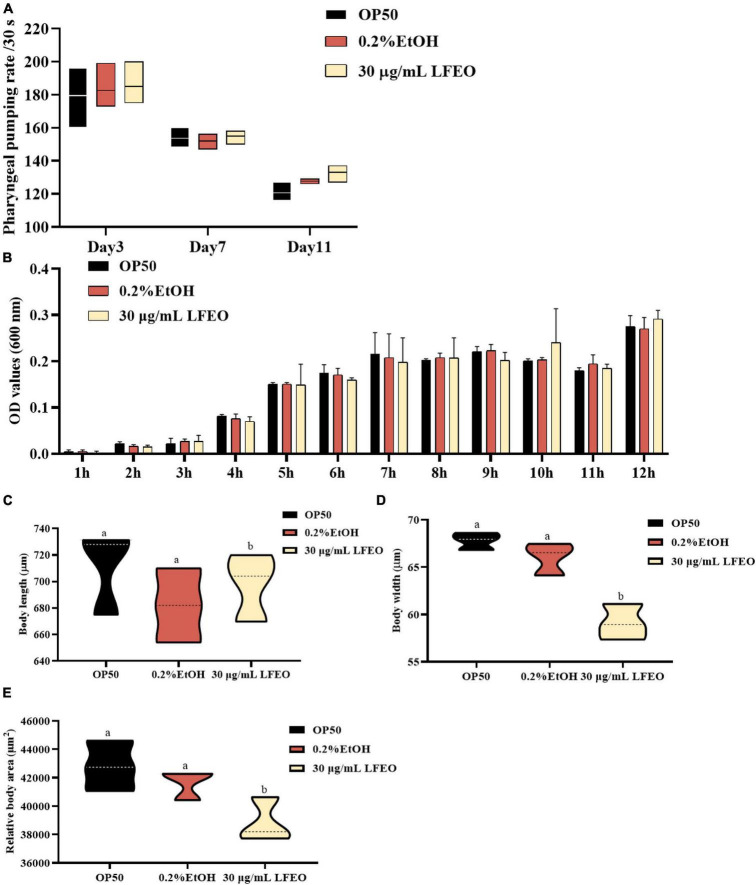
The effect of litchi flower essential oil (LFEO) on energy intake of worms. **(A)** Pharyngeal pumping plays an important role in food intake which is involved in energy intake. The swallowing rate of nematodes at different periods was monitored. **(B)** *E. coli OP50*, as the sole food source for nematodes is associated with energy intake. The effect of LFEO on the incubation of *E. coli OP50* was determined. The effect of LFEO treatment on body length **(C)**, width **(D)**, and area of worms **(E)**. Statistical analysis of the results was done using a one-way ANOVA with Bonferroni tests as *post hoc*comparison; different letters indicate a significant difference, *p* < 0.05; with at least 10 worms per group in panels **(A,C–E)**, and three biologically independent samples in panel **(B)**.

Surplus dietary fat cannot be converted into other nutrients or excreted, and it has to be stored in the form of lipid droplets or produced as energy through oxidation (43). It is well known that fat accumulation can be reduced by decreasing energy intake or increasing energy consumption (44). Accordingly, we further determined whether the LFEO treatment affected the energy metabolism of nematodes. In terms of energy intake, nematodes ingest food through the pharynx, therefore pharyngeal pumping speed, as an indicator of food intake, is associated with energy intake (45, 46). First, the effect of LFEO on the pharyngeal pumping was detected on days 3, 7, and 11, respectively. As shown in Figure 5A, regardless of the duration of LFEO administration, there was no significant change in the pharyngeal pump among the treated groups (*p* > 0.05). As *E. coli* OP50 was the sole food source for the nematodes (47), we further investigated whether LFEO reduced fat accumulation by inhibiting the growth of *E. coli* OP50. The result showed that LFEO did not inhibit *E. coli* OP50 growth condition (*p* > 0.05, Figure 5B). Therefore LFEO did not reduce fat accumulation by inhibiting food intake. Obesity caused by excessive energy intake is characterized by enlarged body shape and size (48). Furthermore, it is reported that energy intake can determine body size (49). Therefore, we measured the body size of nematodes, including width, length, and surface area. As shown in Figure 5C, there was no significant change in body length among these treatment groups (*p* > 0.05). Further, significant reductions were observed in body width and body area in the LFEO-treated group (*p* < 0.05, Figures 5D,E), which may have been caused by the reduction of body fat.

**FIGURE 6:**
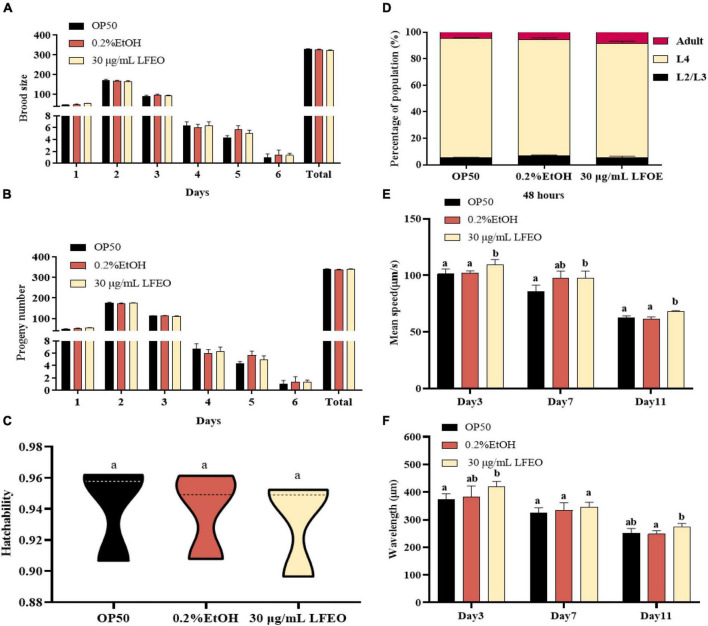
The effect of litchi flower essential oil (LFEO) on energy metabolism of worms. **(A)** Distributions of daily egg production and total egg production of adult nematodes. **(B)** The hatching of laid eggs every day. **(C)** Comparison of hatchability toward nematodes in different groups. **(D)** Determination of LFEO influence on nematode growth rate. Using the WormLab equipment determined the average speed **(E)** and wavelength **(F)** of nematodes that were treated with LFEO for 3, 7, and 11 days, respectively. Statistical analysis of the results was done using a one-way ANOVA with Bonferroni tests as *post hoc*comparison; different letters indicate a significant difference, *p* < 0.05, with at least 10 worms per group.

Increasing energy expenditure, which consists of resting energy expenditure, physical activity, and diet-induced thermogenesis, can reduce fat accumulation (50, 51). In the current study, we mainly studied the effects of LFEO on the two main energy consumption of nematodes, including resting energy consumption (REE) (accounting for 70% of total individual energy expenditure) and exercise consumption (EC) (around 20–35%) (51). The REE is defined as the energy needed to support basic metabolic activity, while physical EC is the most volatile part of energy expenditure (50, 51). Firstly, we separately investigated the growth rate and reproductive capacity of the nematodes, which were associated with REE, to examine whether LFEO treatment increased the REE. It was apparent that LFEO had no significant effect on the growth rate of *C. elegans* (*p* > 0.05, Figure 6D). As shown in Figures 6A–C, no difference was observed among brood size, progeny number, and hatchability, representing the reproductive capacity of *C. elegans* (*p* > 0.05). Secondly, we analyzed the effect of LFEO on the physical EC of the nematodes. The WormLab tracking system and software were utilized to analyze the motricity force of the nematodes, which could capture the motion state of the worms accurately (52, 53). The motricity was evaluated by average speed and wavelength on days 3, 7, and 11, respectively. The wavelength is an important index to evaluate the coordination and balance of the nematodes’ motion, which is positively correlated with the speed of the nematodes’ movement (52, 53). As shown in Figures 6E,F, we found that the average speed and motion wavelength of LFEO-group worms were significantly increased in different life stages compared to the control group (*p* < 0.05). Hence, EC might be involved in LFEO-mediated fat reduction mechanisms.

Litchi flower essential oil significantly reduced the body width and body surface area. This result might be one of the characteristics of the fat deposition reduction mediated by LFEO because LFEO had no negative effects on swallowing, food intake, reproductive ability, body length, growth, and development in *C. elegans*. LFEO might increase energy consumption to reduce fat via accelerating the nematodes’ movement, but had no effect on energy intake.

### Effect of litchi flower essential oil on lipid metabolism pathways of Caenorhabditis elegans

**FIGURE 7:**
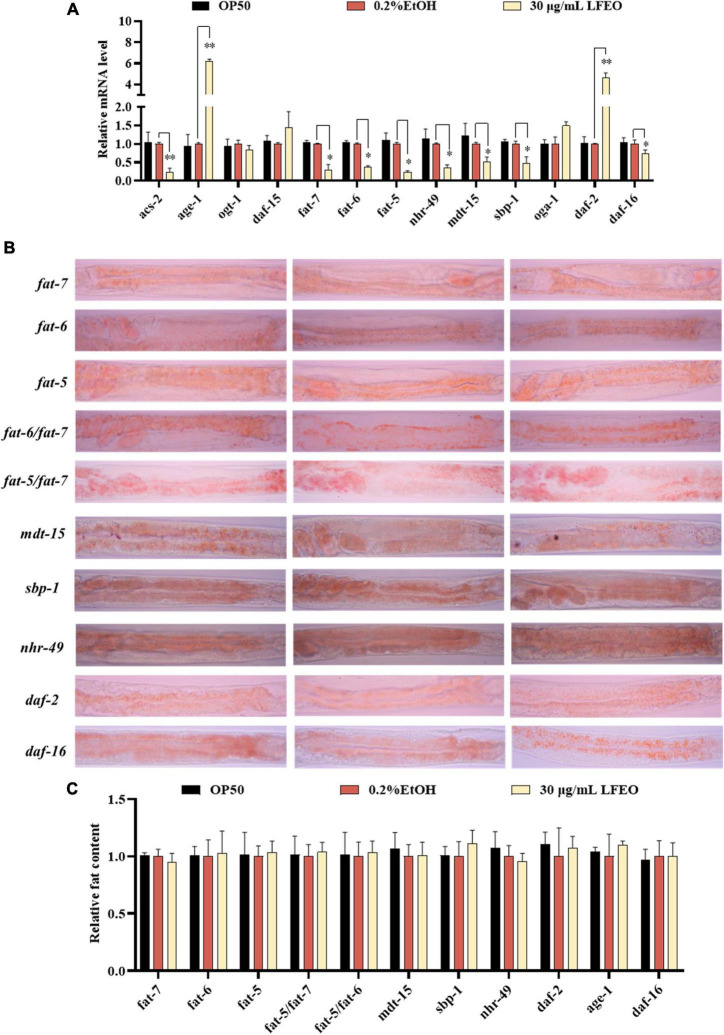
The molecular mechanisms of litchi flower essential oil (LFEO) on fat accumulation and reduction. **(A)** The mRNA level was determined by qRT-PCR and normalized to the expression of *act-1.* **(B)** The representative oil red O (ORO) staining images of the fat content in *age-1*, *daf-2*, *daf-16*, *sbp-1*, *mdt-15, nhr-49*, *fat-5*, *fat-6*, *fat-7*, *fat-5*/*fat-6* double, and *fat-5*/*fat-7* double mutant worms. **(C)** Quantitative analysis of ORO staining intensity using ImageJ software. Statistical analysis of the results was done using a one-way ANOVA with Bonferroni tests as *post hoc*comparison; different letters indicate a significant difference, *p* < 0.05; with three biologically independent samples in panel **(A)** and at least 15 worms per group in panel **(C)**.

In order to further determine the effects of LFEO on lipid metabolism mechanism in nematodes, the mRNA expressions of key genes were investigated in lipid metabolism pathways including the IIS pathway, *sbp-1*/*mdt-15* mediated metabolism pathway, *nhr-49*/*mdt-15* mediated nuclear hormone signaling pathway, target of rapamycin (TOR), and hexosamine metabolism signaling pathway. First, MDT-15 is the homolog of peroxisome proliferator-activated receptor-gamma coactivator-1 α as well as a conserved mediator complex component, which is necessary for regulating FA homeostasis through participating in the expression of genes involved in FA metabolism (30, 54, 55). In this study, we first detected mRNA expression levels of the *mdt-15* gene to determine whether it participated in LFEO-mediated FA metabolism mechanisms. As presented in Figure 7A, LFEO significantly reduced *mdt-15* expression by 49.2%, suggesting that *mdt-15* might be a key regulator of fat reduction induced by LFEO (*p* < 0.05). Moreover, the quantitative results from the *mdt-15* defective mutant’s ORO staining images showed that LFEO failed to reduce intestinal fat accumulation (*p* > 0.05, Figure 7C), which further indicated that *mdt-15* played an important role in the LEFO-mediated lipid-lowering mechanism.

Second, it is known that SBP-1 is the homolog of human SREBP in *C. elegans* and is considered an important transcription factor in maintaining lipid homeostasis (30). SREBP, as a vital transcription factor regulating the metabolism of FA, cholesterol, and other lipids in mammals, is involved in the pathogenesis of various metabolic diseases, especially obesity and obesity-related diseases (56, 57). SBP-1 played a crucial role in lipid homeostasis, which has previously been shown to regulate fat accumulation in worms (58, 59). In addition, MDT-15 can interact directly and specifically with the SBP-1 activation domain (30). Therefore, we investigated whether the transcription level of *sbp-1* was altered by LFEO. As shown in Figure 7A, LFEO significantly down-regulated *sbp-1* gene expression, indicating that *sbp-1* was an essential transcription factor regulating the lipid-lowering effects mediated by LFEO (*p* < 0.05). Subsequently, *sbp-1* defect mutants were used to further verify the conclusion (Figure 7B). The ORO staining results suggested that LFEO had no lipid-lowering effect in the *sbp-1* defect mutants (*p* > 0.05, Figure 7C). This result confirmed our hypothesis that the *sbp-1* gene is an important regulator of the lipid-lowering mechanism mediated by LFEO through SBP-1/MDT-15 signaling pathway.

Third, MDT-15 interacts specifically with NHR-49 which is encoded by the *nhr-49* gene in worms and is the homolog for the peroxisome proliferator-activated receptor α of mammals (60). The gene *nhr-49* acts as a key regulator of fat metabolism and is the upstream target gene of the nematodes’ nuclear hormone signaling pathway (61, 62). As shown in Figure 7A, LFEO down-regulated transcription of *nhr-49* by 65.1% (*p* < 0.05). ORO staining results showed that LFEO-mediated fat reduction was abolished in the *nhr-49* mutant, suggesting that LFEO’s fat-lowering effects were dependent on the NHR-49/MDT-15 signaling pathway (*p* > 0.05, Figure 7C). Furthermore, it has been reported that transcriptional factors *mdt-15*, *sbp-1*, and *nhr-49* genes could regulate the expression of SCDs genes (63). SCDs is a key enzyme in the neolipid pathway, which is responsible for forming monounsaturated FA from saturated FA by catalyzing the insertion of double bonds into the ninth carbon of the saturated C16 and C18 substrate (26). Since the above results suggest that SBP-1/MDT-15 and NHR-49/MDT-15 signaling pathways might be potential factors for lipid reduction induced by LFEO, we further explored the role of monounsaturated FA-mediated pathways in the lipid reduction mechanism. There are three important genes encoding SCDs in nematodes: *fat-5*, *fat-6*, and *fat-7*, respectively. FAT-6 and FAT-7 easily desaturated stearic acid (C18:0) and showed less activity on palmitic acid (C16:0) (26). Conversely, another desaturating enzyme, FAT-5, readily desaturates palmitic acid (C16:0). As displayed in Figure 7A, the expression of the *fat-5*, *fat-6*, and *fat-7* genes were decreased by 76, 63.4, and 70.3%, respectively, after the treatment with LFEO (*p* < 0.05). Furthermore, we detected the effect of LFEO on fat accumulation in the single (*fat-5*, *fat-6*, and *fat-7*) and double mutants (*fat-5*/*fat-6* and *fat-5*/*fat-7*) (Figure 7B). LFEO had no effects on lipid storage in single-mutant and double-mutant strains, suggesting LFEO-induced fat reduction depended on *fat-5*, *fat-6*, and *fat-7* (*p* > 0.05, Figure 7C).

Furthermore, the nuclear hormone receptor NHR-49 has been shown to regulate FA β-oxidation, FA desaturation, and FA binding/transportation in *C. elegans* (62, 63). As the *nhr-49*’s target gene, *asc-2* is an essential gene for regulating mitochondrial β-oxidation, encoding mitochondrial acyl-CoA synthetase (61). Curiously, LFEO treatment significantly decreased the mRNA level of *acs-2* (*p* < 0.05, Figure 7A). We speculate that the expression of *the acs-2* gene was suppressed by *nhr-49* to inhibit β-oxidation activity and this was ultimately reflected in the reduction of lipid synthesis in *C. elegans*.

Moreover, the IIS (insulin/insulin growth factor signaling) is an evolutionally conserved signaling pathway that is involved in the regulation of lipid metabolism (64, 65). The DAF-2 (insulin receptor-like protein), AGE-1 (phosphatidylinositol-3-OH kinase catalytic subunit], and DAF-16 (forkhead box O transcription factor) are the most critical components responsible for regulating fat metabolism involved in the IIS pathway (64, 65). In *C. elegans*, the IIS pathway is initiated by the activation of DAF-2, which leads to phosphorylation and the activation of a series of downstream protein kinases including phosphoinositide 3-kinase (PI3K)/AGE-1, 3-phosphoinositide-dependent protein kinase-1, and protein kinase B to inactivate the DAF-16 (64, 65). As shown in Figure 7A, LFEO significantly increased the expression of *daf-2* and *age-1*, while down-regulating *daf-16* gene expression, suggesting that *daf-2*, *age-1*, and *daf-16* might be involved in LFEO-mediated lipid lowering (*p* < 0.05). We further evaluated the effect of LFEO on fat accumulation in *daf-2*, *age-1*, and *daf-16* mutants to confirm the results (Figure 7B). The ORO results showed LFEO had no effect on the intestinal fat deposition of the three mutants which confirmed our presupposition (*p* > 0.05, Figure 7C). Moreover, it has been reported that *daf-16* and *daf-2* could regulate the expression of *fat-5* (66). Therefore, we assumed that LFEO mediated lipid-lowering mechanism might, *via* the DAF-2/ IIS signaling pathway, regulate *fat-5*, *fat-6*, and *fat-7* expression. Moreover, the key pathways of nematode lipid metabolism are TOR and the hexosamine metabolism signaling pathway (63, 67). For instance, the DAF-15 (Raptor N domain-containing protein) acts as a mammal homolog of a protein kinase associated with TOR, regulating cell growth and proliferation by integrating nutrients, energy, and growth factors (63). The related OGT (Protein O-GlcNAc transferase) and OGA (O-GlcNAc selective *N*-Acetyl-beta-D-glucosaminidase) regulate cellular energy levels through mediating amino hexose signaling (63). To detect whether LFEO regulates TOR and the hexosamine metabolism signaling pathway, the mRNA expressions of target genes *daf-15*, *ogt-1*, and *oga-1* of the above proteins were assayed in worms. The results revealed that LFEO did not change the expressing levels of *daf-15*, *ogt-1*, and *oga-1* in *C. elegans* (*p* > 0.05, Figure 7A), which suggested that TOR and the hexosamine metabolism signaling pathway might not be involved with LFEO-mediated lipid lowering.

**FIGURE 8:**
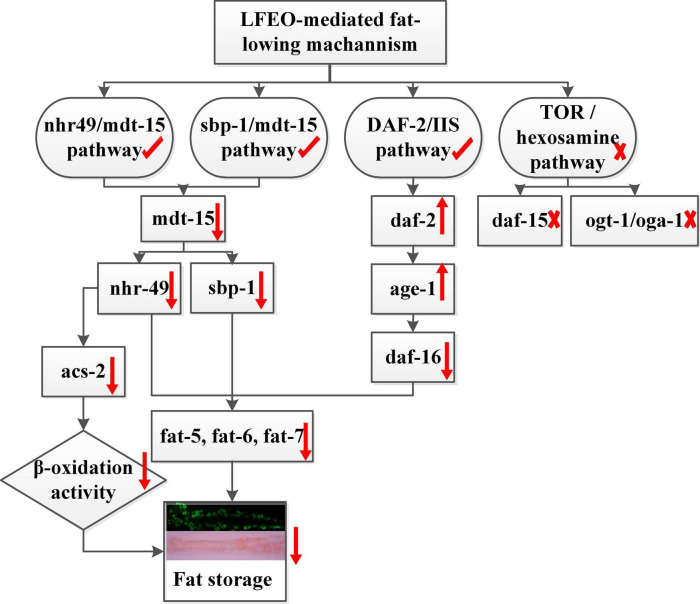
The molecular mechanism of litchi flower essential oil (LFEO) mediated lipid-lowering effects.

Given the effects of LFEO on fat accumulation in *C. elegans*, we further explored several possible mechanisms, including the DAF-2/IIS pathway, *sbp-1*/*mdt-15* mediated metabolism pathway, *nhr-49*/*mdt-15* mediated nuclear hormone signaling pathway, TOR and the hexosamine metabolism signaling pathway. In conclusion, the current study has shown that LFEO significantly reduced fat accumulation in *C. elegans* by reducing fat synthesis and increasing lipolysis. As shown in Figure 8 we proposed that LFEO-mediated molecular mechanisms for reducing fat accumulation might, via *sbp-1* and *nhr-49* regulated by *mdt-15*, and *daf-16* adjusted by *daf-2* and *age-1*, co-target SCDs genes *fat-5*, *fat-6*, *fat-7* in *C. elegans*. Moreover, LFEO might reduce the β-oxidation activity through *nhr-49* inhibiting the expression of *acs-2* genes in response to the promotion of lipolysis caused by the NHR-49/MDT-15 signaling pathway.

In short, the CPTD developed by our research group was applied to extract essential oils from litchi flowers to solve the pollution and environmental deterioration caused by the large number of abandoned litchi flowers. This is of great significance in promoting the development of a circular economy and realize the 2030 United Nations SDGs. Consistent with the theme of SDG 2 (“improving malnutrition”), which includes obesity caused by overnutrition (68), the study on the lipid-lowering activity of LFEO provides a new idea for the development of lipid-lowering products. Moreover, we will further explore the application of LFEO in food safety preservation in the future since essential oils are extensively used as natural additives in food security and food preservation (69). This is consistent with the emphasis on “realizing food security” in SDG 2 (68).

## Conclusion

In summary, our research illustrated that LFEO could significantly reduce the fat accumulation and TG content of nematodes in normal, glucose-feeding, and high-fat conditions. LFEO had a significant effect on reducing both the size and number of lipid droplets in ZXW618. In terms of energy metabolism, LFEO treatment had no effect on energy intake but might increase energy consumption by expediting the average speed and wavelength. The decrease in body width and body area was shown to be induced by the lipid reduction due to LFEO because LFEO had no side effects on other physiological activities. Furthermore, the lipid-lowering effects mediated by LFEO were shown to be involved with several signaling pathways, including the IIS pathway, *sbp-1*/*mdt-15* mediated metabolism pathway, and the *nhr-49*/*mdt-15* mediated nuclear hormone signaling pathway. Moreover, LFEO may inhibit the expression of *acs-2* genes through *nhr-49* to reduce the β-oxidation activity. The research provides a new idea for recycling LFEO from litchi flowers at large-scale production and exploiting LFEO’s lipid-lowering properties. Finally, this study contributes to extending the application of LFEO and is of great significance for the development of LFEO.

## Data availability statement

The original contributions presented in this study are included in the article/Supplementary material, further inquiries can be directed to the corresponding authors.

## Author contributions

YCh: formal analysis, investigation, writing – original draft, and writing – review and editing. QQ: investigation and data curation. JL: project administration, visualization, and investigation. YD: resources. CL: visualization and investigation. HC and YCa: methodology and writing – review and editing. YJC and ZS: conceptualization, methodology, writing – review and editing, and funding acquisition. All authors contributed to the article and approved the submitted version.

## Abbreviations

*C. elegans*: *Caenorhabditis elegans*
CPTD: continuous phase transformation device
DHS: dehydrogenase
*E. coli OP50*: *Escherichia coli OP50*
EC: exercise consumption
FA: fatty acids
GC-MS: gas chromatography-mass spectrometry
IIS: insulin/IGF-1 signaling
LFEO: litchi flower essential oil
MDT-15: mediator-15
NHR: nuclear hormone receptor
ORO: oil red O
qRT-PCR: quantitative reverse transcription-polymerase chain reaction
SDGs: sustainable development goals
REE: resting energy consumption
SREBP: sterol response element binding protein1
SCDs: stearoyl-CoA-Δ9-desaturases
TG: triglyceride
TOR: target of rapamycin
TGF-β: transforming growth factor-β.
