# Supplementary material for: Litchi flower essential oil balanced lipid metabolism through the regulation of DAF-2/IIS, MDT-15/SBP-1, and MDT-15/NHR-49 pathway
Source: Front Nutr. 2022 Oct 19;9:934518. doi: 10.3389/fnut.2022.934518 (PMC9627157; doi:10.3389/fnut.2022.934518)
Supplement: Supplementary file 1 [file Table_1.pdf]

Supplementary materials

Primer sequences used for qRT-PCR

Yun Chen[a,b], Qiao Qin[a,b], Jingrui Luo[a,b], Yusi Dong[a,b], Chunxiu Lin[a,b], Houbin Chen[a,b], Yong Cao[a,b], Yunjiao Chen[a,b]*, Zuanxian Su [a,b]*

[a] South China Agricultural University, Guangzhou 510640, Guangdong, China.

[b] Guangdong Laboratory for Lingnan Modern Agriculture, Guangzhou 510640, Guangdong, China.

Corresponding Authors

*Email: yunjiaochen@scau.edu.cn (Yunjiao Chen), zuanxsu@scau.edu.cn (Zuanxian Su); Telephone: +86-20-85286234(YC)/+86-20-85280231(ZS).

Table. S1 Primer sequences used for qRT-PCR

Gene	Forward primer	Reverse primer
act-1	CAAGGAGTCATGGTCGGTATG	TCAATTGGGTACTTGAGGGTAAG
mdt-15	CGTAGCAACAACACAGGCATCAAC	AACAGCAGCAGTGGCAGAAGC
nhr-49	GCCAGATGACGCACCCACAAG	GAATCCACCTCTGCCAATCCGAAG
sbp-1	AATCTGGGTTTGGCGGTTGGC	CGAGCGACTTCTTTGTGTGAATGC
acs-2	ATGCGCTCAATCCTCGTATC	GATGGGTCAGGTGATGAGAATC
daf-2	CCACGACGACGAGCACATCAC	GGCGGGTTTTCCTCATAGCAGTC
age-1	GATTGCTGGACACGGACGGAAC	GATCCTTTGCCATTCTCGGTCTCC
daf-16	GCTCTGCTGAAAGTCCCATCCTG	CCGTGAATCCGTTCCAACCGTAG
fat-5	GCCCTCTTCCGTTACTGCTTCAC	CTTCTCCGACTGCCGCAATAGATG
fat-6	TCGGAGAGGGAGGTCACAACTTC	CGGTCGTAGACAAGTCCAAGAGC
fat-7	ATCGTTGCCATCACAAGTGGACTG	TTACGCACAAGAAGCCATCCCATG
ogt-1	TTCACATGCGGCTCGGATTGC	GATCAGCGTGGATGAGTGGTGTC
oga-1	GCACAAGTTCACATCGCCAATACC	ACCGCTCGTGACTCGCAATATTC
daf-15	GCGTTGTCTCCTGGTAGTTCGTTC	TCGTCGTCATCGGAAGAGTCGTC
